# Reprogramming of Thyroid Cancer Metabolism: from Mechanism to Therapeutic Strategy

**DOI:** 10.1186/s12943-025-02263-4

**Published:** 2025-03-11

**Authors:** Yuxuan Wan, Guoqing Li, Gaoyuan Cui, Saili Duan, Shi Chang

**Affiliations:** 1 Department of General Surgery, Xiangya Hospital Central South University, Changsha, 410008 Hunan People’s Republic of China; 2 Xiangya School of Medicine, Central South University, Changsha, 410008 Hunan People’s Republic of China; 3 Department of Cancer Biology, University of Michigan, Ann Arbor, MI 48109 USA; 4 National Clinical Research Center for Geriatric Disorders, Xiangya Hospital, Changsha, 410008 Hunan People’s Republic of China; 5 Clinical Research Center for Thyroid Disease in Hunan Province, Changsha, 410008 Hunan People’s Republic of China; 6 Hunan Provincial Engineering Research Center for Thyroid and Related Diseases Treatment Technology, Changsha, 410008 Hunan People’s Republic of China

**Keywords:** Thyroid cancer, Metabolism, Genetics, Carcinogenesis, Diagnosis, Targeted therapy

## Abstract

Thyroid cancer as one of the most prevalent malignancies of endocrine system, has raised public concern and more research on its mechanism and treatment. And metabolism-based therapies have advanced rapidly, for the exclusive metabolic profiling of thyroid cancer. In thyroid cancer cells, plenty of metabolic pathways are reprogrammed to accommodate tumor microenvironment. In this review, we initiatively summarize recent progress in the full-scale thyroid cancer metabolic rewiring and the interconnection of various metabolites. We also discuss the efficacy and prospect of metabolic targeted detection as well as therapy. Comprehending metabolic mechanism and characteristics of thyroid cancer roundly will be highly beneficial to managing individual patients.

## Introduction

Metabolism includes a series of chain reactions converting nutrients intake into energy as well as producing structural components such as protein and nucleic acids [1]. It is essential for every cell of living organisms to maintain basic functions, which rely on the balance between anabolism (synthesis) and catabolism (degradation) [2]. We usually call glucose, lipids and amino acids “fuels” in our body because they can be oxidated and produce a large amount of adenosine 5’-triphosphate (ATP) for energy supply [3, 4]. Among the energy-supplying substances, glucose and lipids serve as the major sources of energy in our body, and in most cases, glucose oxidation keeps the priority [5]. These principles guarantee that cellular metabolism could generate enough ATP with the maximum efficiency. As is widely proved, energy demands vary by organs and tissues, as well as their metabolic status. For instance, the brain energy consumption accounts for over 20% of the total in order to coordinate with neural activities [6–8] and the skeletal muscles can quickly activate metabolic pathways for ATP synthesis during exercise [9]. In cancer cells, the overall metabolism is reprogrammed so significantly that the malignantly mutated and proliferating cells are able to survive in a low-nutrient and acidic milieu [10]. They form a new metabolic network, in which biosynthesis, electron carrier regeneration and metabolic control are comprehensively redirected, to support the high-speed growth of cancer cells and their unique behavior of metastasis [11, 12]. These altered metabolic pathways involved with glucose, lipids, amino acids, and nucleotides, among others, allow cancer cells to acquire nutrients quite a lot more than normal cells, and to some extent, break the barrier between scarce nutritional sources and high energy demands [13, 14]. This feature exists in all kinds of cancers, including thyroid cancer (TC), one of the most common endocrine malignancies worldwide and has increased in number markedly in the past decades [15, 16]. TC is derived from follicular thyroid cells covering papillary thyroid cancer (PTC) and follicular thyroid cancer (FTC), and a small portion from parafollicular C cells, which mainly contains medullary thyroid cancer (MTC) [17, 18]. Though there’s evidence suggesting the mortality of TC remains stably flat [19, 20], several types of TC such as anaplastic thyroid cancer (ATC) and metastatic poorly differentiated thyroid cancer (PDTC) show poor overall survival even with multimodal therapy [21]. The prognosis of TC is closely connected to the situation of invasion and metastasis [22] as well as the mutation sites [23], requiring continuous research on new targets for detection and therapy.

Thyroid as an endocrine organ displays a specific metabolic landscape, making TC distinct from other malignancies. Hence, aiming at the aberrant metabolism, which is identified as a hallmark of cancer [24], shows great potency in the individualized management of TC and provides methods for early diagnosis to improve survival rates. In the present review, we summarize how the metabolism is reprogrammed in TC and discuss how metabolic targets could be exploited for diagnosis and treatment.

## Mechanisms of metabolic reprogramming in TC

**Fig. 1:**
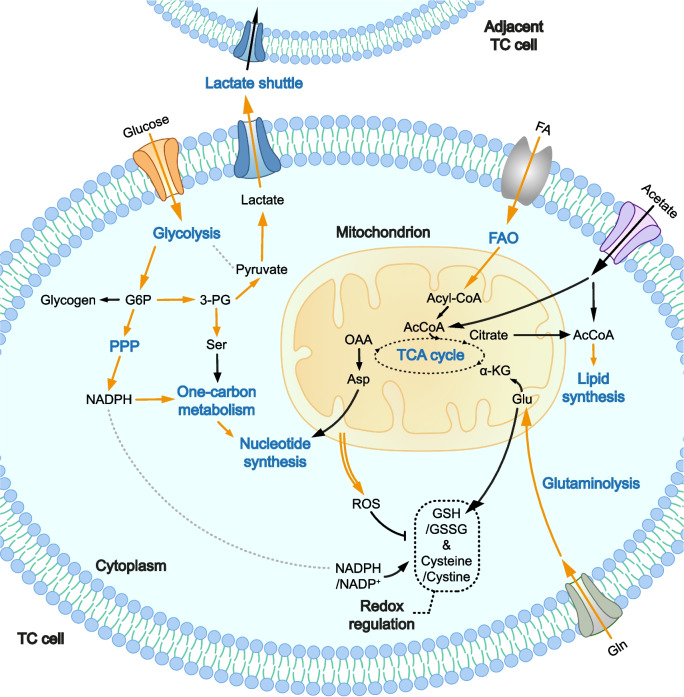
Overview of metabolic intersections in TC cells. The metabolic pathways in TC cells have an intricate interplay of intermediate products, metabolites and enzymes. By this closely connection covering glycolysis, PPP, one-carbon metabolism, the TCA cycle, lipid and amino acid metabolism and others, TC cells are able to optimize the holistic metabolic pattern to the maximum extent. The TCA cycle as the center of cell metabolism, mutates significantly for faster energy production. Since quite more lactate molecules are generated, the peculiar lactate shuttle is established, transmitting fresh fuel supply to the neighboring TC cells. With this subtle design, TC can progress rapidly. Abbreviations (not mentioned in the text): 3-PG, 3-phosphoglycerate; Ser, serine; OAA, oxaloacetate; α-KG, α-ketoglutarate; Asp, aspartate; Glu, glutamic acid; Gln, glutamate

It has been a century since Otto Warburg first revealed that tumor tissues convert much more glucose into lactic acid (LA) than normal tissues [25], which has broken new ground in the exploration of cancer metabolism. He put forward the concept of aerobic glycolysis where tumor cells choose to generate ATP in an insufficient way, but benefit from it in general [26]. From then on, the relevant molecular variations, altered pathways and their relationships with tumor initiation and progression have been discovered [27], giving rise to therapeutic strategies targeting aberrant metabolism [28]. Digging deeper into the mechanism of metabolic alterations in malignancies, it is clearer that cancer should be considered as a metabolic disorder together with genetic disease [29], and thus cancer metabolism should be observed from a holistic view. The reprogramming of metabolism in TC is an intricate process where glucose, lipid, amino acid, and nucleotide metabolisms are intertwined. For example, changes in glucose metabolism can influence the intermediates for lipid synthesis, thereby affecting the overall metabolic balance of TC cells. Understanding these interconnections is crucial for a comprehensive view of TC metabolism (Fig. 1). The intersected metabolic pathways are designed so exactly that the nutrient acquisition and energy biosynthesis could perfectly meet the insatiable demands of tumor cells [30]. It can be speculated that once a single metabolite or enzyme shifts in structure, the related pathways could be redirected, causing specific metabolic imbalance. Consequently, understanding the mechanism of reprogrammed metabolism in TC is vital for further breakthroughs in treatment.

### Glucose metabolism in TC

**Fig. 2:**
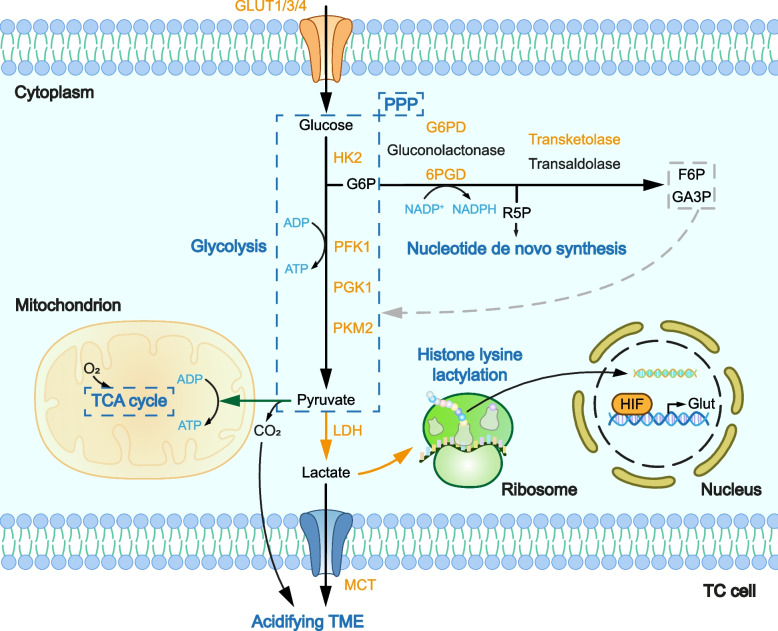
Glucose metabolism in TC

Ever since Warburg laid the foundation for the integral view of cancer biology [31] in which cancer metabolism shares the equal significance with signal transduction and transcription [32], the altered glucose metabolism has been the most investigated [33] and proven to play an important role in TC development [34]. The cancer cells prefer converting glucose into LA than letting pyruvate enter the tricarboxylic acid (TCA) cycle, which is coupled with oxidative phosphorylation (OXPHOS) [35]. This allows glycolytic intermediates to be directed to the pentose phosphate pathway (PPP) for production of NADPH and other biomass synthesis [36, 37]. With specific purposes, cancer cells manipulate the glucose metabolism and take full advantage of it. Figure 2 provides a comprehensive view of altered glucose metabolism based on the available evidence in TC.

After glucose is absorbed by TC cells via the overactive GLUTs, it enters glycolysis and generates ATP at a high speed. Pyruvate is mostly converted into lactate rather than transferred into mitochondria. The accumulated CO_2_ and lactate consistently acidify TME, sending chemical signals to the ambient milieu. Lactate also participates in histone modifications, which may influence transcriptional activities. Additionally, by elevating PPP-related enzymes, nucleotides are more accessible, facilitating TC cell proliferation.

### Glucose uptake

Compared with normal cells, cancer cells show a large appetite for glucose, since glucose is the main source of energy [35]. To realize the unstoppable growth, the first step is to obtain great more glucose, which is supported by glucose transporters (GLUTs) [38, 39] encoded by solute carrier family 2 (*SLC2*) genes with GLUT1-14 (proteins) corresponding to *Slc2a1-14* (genes) [40]. GLUTs transfer glucose from extracellular matrix (ECM) into cytoplasm for subsequent reactions [41], and the dysregulation of these transporters is closely related to cancer activities [42]. Previous research has revealed the upregulation of GLUT1, GLUT3 and GLUT4 in TC, and poorly differentiated types (anaplastic) mainly express high level of GLUT1, while well-differentiated types (follicular and papillary) have stronger GLUT3 and GLUT4 expressions than GLUT1 [43], indicating that the glucose uptake vary by malignant degree. Targeting specific types of GLUT could potentially be an individual-based treatment. In a study evaluating the relationship between *SLC2* expressions and prognosis, overexpression of *SLC2A1, SLC2A3, and SLC2A14* decreases the survival in PTC [44], which provides evidence for the adverse effect of upregulated GLUTs in TC. And high level of GLUT1 can be attributed to the downregulation of long non-coding RNA (lncRNA) *NBR2* [45], fat-mass and obesity-associated protein (FTO) [46] and oncosuppressor phosphatase and tensin homolog (PTEN) [47], as well as upregulation of SIRT6 [48, 49] and afamin [50], and *BRAF* mutation [51]. Moreover, study has found the downregulation of GLUT1 in TC metastasized from other sites rather than primary TC, which implies the different metabolic features of TCs from diverse sources [52]. Except for GLUT1, the regulation of GLUT3 has also been reported. For instance, sineoculis homeobox homolog 1 (Six1) overexpression lifts GLUT3 expression in TC [53]. Estrogen (E2)-related DNA methyltransferases 3B (DNMT3B)-mediated family with sequence similarity 111 member B (*FAM111B*) methylation and upregulation of *circRAD18* were observed to promote PTC glucose uptake as well, indicating the overexpression of GLUTs [54, 55]. Regulation of other types of GLUT in TC has not been illustrated in detail. From the subtle strategy that TC cells choose, we can infer that although they don’t produce ATP in the most efficient way, they absorb masses of nutrients especially glucose to compensate, making ATP production the biggest amount within the shortest time for their invasive ambition. Besides, a new concept on glucose-to-lymphocyte ratio was proven to predict central lymph node metastasis in PTC patients with type 2 diabetes mellitus [56]. This opens new ideas that high blood glucose may provide even more adequate energy for cancer cells, facilitating TC metastasis. In short, TC cells aim at “quantity” but not “quality” in glucose utilization, which can be extended to other cancer types.

### Glycolysis and lactate production

#### Different behaviors of normal and cancer cells in glucose processing

For both normal cells and cancer cells, they absorb glucose to gain ATP from the oxidation reactions. Normal cells choose aerobic oxidation of glucose, while cancer cells prefer anaerobic glycolysis [57]. This conclusion also holds true in TC. For example, as a transcriptional coactivator, peroxisome proliferator-activated receptor γ coactivator 1α (PGC1α) has been reported to be downregulated in TC cells, thereby inhibiting oxidative phosphorylation and inducing a glycolytic phenotype. Specifically, the downregulation of PGC1α leads to increased glucose uptake and lactate production in TC, while ATP generation and oxygen consumption are decreased [58]. The sharing first step of two types of oxidations is glycolysis, which consists of several reactions decomposing glucose into pyruvate [59]. Whereafter, pyruvate arrives at the fork junction. It is supposed to enter the TCA cycle as acetyl coenzyme A (AcCoA) after the oxidative decarboxylation by pyruvate dehydrogenase in normal metabolic status [60], but it turns out to be priorly transferred into LA in cancer cells and then shuttled to adjacent cells or stroma [61]. In physiological pH range of body fluid, over 99% of LA is dissociated into lactate anions (La^−^) and protons (H^+^) [62], so lactate (not LA) is then transported into the extracellular space. The shifting is determined by a series of enzyme deformation and abnormal accumulation of intermediates or products.

#### Rearranged key enzymes of glycolysis in TC

Glycolysis is under the regulation of numerous enzymes, which catalyze the sequential reversible or irreversible biochemistry reactions [63]. The highly heterogenous enzymes in glycolysis facilitate cancer cells to better survive in different environments through multiple smart disguises such as expressing isoforms and making posttranslational modifications [64]. It is well accepted that gene expression and metabolic processes offer feedback to each other, which manifests a mutual regulation [65]. The three rate-limiting enzymes in glycolysis, covering hexokinase 2 (HK2), phosphofructokinase 1 (PFK1) and pyruvate kinases type M2 (PKM2) [66], play an important role in the glycolytic upregulation in TC. In TC, previous study has shown that acidic nuclear phosphoprotein 32 family member E (ANP32E) promotes glycolysis via AKT/mTOR/HK2 pathway [67]. Suppression of FTO [46], and overexpression of afamin [50] and SIRT6 [48] also elevate HK2 transcription. In TC, there is a universal PKM2 upregulation [68], involving *circNRIP1* activated by alkB homolog 5 (ALKBH5) decrease [69], AMP-activated protein kinase (AMPK) [70], downregulated micro RNAs (miRNAs) such as *miR-148a* and *miR-326* [71], *BRAF* mutation [72], as well as overexpression of phosphoribosyl pyrophosphate amidotransferase (PPAT) [73] and SIRT6 [48]. The detailed regulation of PFK1 in TC has not been illustrated yet, but the increase of this enzyme regulating the first committed step of glycolytic pathway is clear [74, 75].

#### Lactate metabolism in TC

Lactate as the product of glycolysis, had long been considered as a metabolic waste [76] until its essential functions in serving as a regulatory molecule in signaling bridging, posttranslational modifications and pathological progress of cancer were uncovered [77]. The alterations of lactate metabolism in TC aligns with patterns observed across pan-cancer largely. Firstly, lactate dehydrogenase (LDH) is significantly elevated in TC [78], leading to an intensified lactate production driven by glycolysis [79]. Then lactate shuttle occurs, cancer cells secrete high levels of lactate into microenvironment through mono-carboxylate transporters (MCTs) [80], and the neighboring cancer cells absorb them as new energy substrates [61]. In a glucose-deprived tumor microenvironment (TME), which is generally seen when nutrients are depleted, high level of lactate is converted into pyruvates (LDH-catalyzed reverse reaction) and then cancer cells can utilize pyruvate for the TCA cycle through the action of pyruvate kinase [81]. In TC, pyruvate kinase M2 (PKM2) is significantly overexpressed and, as a key driver of glycolysis, confers a selective growth advantage to PTC cells [72]. Lactate shuttle testifies that lactate as an energy vehicle, transfers energy from one group of cancer cells to another [82] and participates in realizing the redox balance [83]. This system is so ingeniously designed that the lactate production remains high-speed owing to the constant export of lactate, which promotes the ongoing of glycolysis (removing products keeps the reaction forward), and the reuse of lactate diminishes energy waste and thus aids the high-flux metabolism of cancer cells. Except for energy carrier, lactate takes part in epigenic reprogramming and signal transduction as well, which regulates the various events in the TME [84]. It is reported that in ATC, lactate accumulation promotes globally protein lactylation, especially the histone lysine lactylation, activating the expression of multiple essential genes involved in ATC including *CTGF, CCNE1, CDK1, KLF2, IL1B, and AURKB* [85]. However, the non-histone lactylation exists broadly but lacks attention [86, 87]. Furthermore, the accumulated lactate itself has an effect on changing TME and induces cancer cells reprogramming themselves to adapt to it. In the microenvironment of TC, lactate accumulation reduces the pH, leading to local acidosis. Simultaneously, excessive lactate promotes the epithelial-mesenchymal transition process by enhancing H3K27 acetylation of related genes [88].

Above all, lactate can affect the cancer metabolism greatly by multiple tactics from regulating gene expression and metabolic pathways of cancer cells to influencing the overall TME. Therefore, lactate metabolism is undeniably important in TC. Multiple molecules are upregulated or downregulated with lactate metabolism changing through diverse pathways.

#### PPP

PPP is a specialized energy metabolism pathway, with glucose-6-phosphate dehydrogenase (G6PD) serving as its rate-limiting enzyme. PPP offers R5P as the substrate for nucleotide synthesis, sugar phosphate as the precursor for amino acid synthesis and NADPH as the reducing molecule for redox homeostasis [89]. With its pivotal effects in cell proliferation, PPP is the leading upregulated glucose shunt in cancer cells [90], which highly demand rapid biosynthesis. G6PD catalyzes the dehydrogenation of glucose-6-phosphate (G6P), playing a pivotal role in regulating the flux through the PPP [91]. It has been widely proved that the overexpressed G6PD increases cell proliferation and adaption in various types of cancers, and TC is no exception [92]. Previous research has established that G6PD mutation exists in individuals with both benign thyroid lesion in one lobe (BTG) and PTC, and G6PD may be related to BTG-to-PTC transformation [93]. Inhibition of G6PD and transketolase also shows potential clinical value by leading to reactive oxygen species (ROS)-mediated apoptosis in TC cells [94]. Another critical enzyme of PPP, 6-phosphogluconate dehydrogenase (6PGD), is upregulated in ATC and inhibition of 6PGD is proven to disrupt the doxorubicin-resistant situations in ATC cells [95]. Little research has focused on the detailed remodeled PPP in TC, but without doubt taking PPP as the entry point has a hopeful prospect.

#### Lipid metabolism in TC

**Fig. 3:**
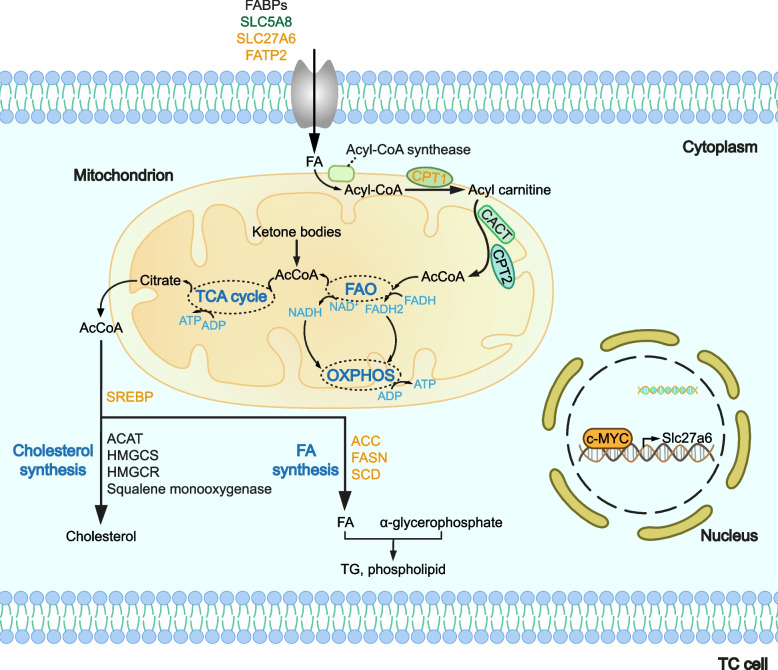
Lipid metabolism in TC. To properly utilize FAs of different chain lengths, TC cells redistribute carriers with their respective capabilities. With the aim of replenishing energy supply approaches, the upregulated CPT1 transports more FA-generated acyl-CoA into the mitochondrion, and subsequently performs FAO and enters the TCA cycle, not wasting any component of a cell. And citrate from the TCA cycle can be translocated to the cytoplasm and then generate considerable cholesterol, TG and phospholipid, which are essentials in TC cells. Abbreviations: ACAT, cholesterol acyltransferase; HMGCS, 3-hydroxy-3-methylglutaryl-CoA synthase; HMGCR, 3-hydroxy-3-methylglutaryl-CoA reductase

Like most cancer, TC is also a comprehensive metabolic syndrome, so there are extensive variations in the entire metabolic routes. Lipids as the second most consumed energy source in most tissues, play a major part in energy storage, constituents of cell membrane and signal transduction [96]. Classified by chemical structures, lipids can be divided into fat, lipoid consisting of steroid (majorly cholesterol), phospholipid, and glycolipid [97–99]. A Mendelian randomization study targeting Italians has shown that disordered serum lipids, including total cholesterol, high-density lipoprotein, apolipoprotein B, and the ratio of apolipoprotein B to apolipoprotein A1, are associated with the risk of DTC [100]. Additionally, patients with more aggressive TCs (high risk PTC or ATC) exhibited high low-density lipoprotein (LDL) level, which might be attributed to the elevated expression of LDL receptors in TC cells. Subsequently, LDL promoted the proliferation and invasion of TC cells [101]. Similarly, as the fundamental component of cholesterol and other lipids, it is demonstrated that fatty acid (FA) is also involved in TC progression through increased uptake by TC cells [102]. There is evidence to contribute this idea. For example, it has been demonstrated that compared with adjacent normal tissues, the monounsaturated fatty acids (MUFAs) level in TC tissues is significantly elevated in TC [103], along with that, the serum level of MUFAs is decreased [104]. In addition, researchers have found that several FA receptors are upregulated, suggesting the enhanced FA uptake in TC. For instance, FATP2 as long-chain FA transporters, and lipoprotein lipases are proven to increase in PTC tissues [105]. High SLC27A6 expression expedites TC progression via upregulating c-MYC, adding to the transport of long-chain FAs [106]. And there is evidence showing that SLC5A8 as a Na^+^-coupled short-chain FA transporter, is downregulated in classical form PTC, which is related to *BRAF* mutations [107, 108]. The different variations in FA transporters imply that TC cells possibly tend to derive certain kinds of FAs as the energy fuel. The increased expression of FA receptors in tumor cells is also associated with the crosstalk between adipocytes and tumor cells within the tumor microenvironment. The hydrolysis of stored triglycerides (TGs) in adipose tissues (mainly white adipose tissues) under the catalyzation of lipase release FAs and glycerols [109]. Under this circumstance, adipocytes are activated and the neighboring cancer cells then take in FAs as the energy fuel via multiple FA transporters [110]. However, the thyroid gland contains a minimal number of adipocytes, thus it is waiting for further research into its mechanism. Interestingly, blood TG/glucose index is proven to be higher in patients with malignant TC than healthy controls, suggesting the altered metabolic process of glucose and FA in TC [111]. In addition, how TC cells influence adipose cells and take advantage of them needs to be studied. See Fig. 3 for a detailed information of lipid metabolism rewiring as well as the links between lipids and other metabolites.

#### FA β-oxidation

In a physiological manner, FAs serve as substitute fuels when there’s a glucose shortage. To acquire energy, FA activation is a must, where FAs are converted into acyl-CoA by the acyl-CoA synthetase on the outer mitochondrial membrane, followed by entering mitochondria with the assistance of carnitine palmitoyltransferase 1 (CPT1) on the outer mitochondrial membrane together with carnitine-acylcarnitine translocase (CACT) and carnitine palmitoyltransferase 2 (CPT2) on the inner membrane [112]. The oxidation of FAs, which is commonly known as FA β-oxidation (FAO), includes several basic steps in the mitochondrial matrix. Acyl-CoA is degraded into AcCoA, FADH_2_ and NADH by certain enzymes, and the products are to enter the oxidative respiratory chain (also known as the electron transport chain) or the TCA cycle for ATP synthesis [113]. Among processes above, CPT1 is the critical rate-limiting enzyme in FAO [114]. Although little evidence shows that FAO-associated processes are significantly reprogrammed, there are observations indicating the CPT1A and CPT1C upregulation in TC [105, 115], suggesting the enhanced FAO in TC cells. By enhancing the transport of acyl-CoA into mitochondria for oxidation, TC cells can utilize fatty acids as an alternative energy source more efficiently, highlighting the importance of lipid metabolism in supporting the growth and survival of TC cells. Moreover, lncRNA *SOCS2-AS1* is proven to promote p53 degradation and FAO rate, which boosts PTC progression [116].

#### Ketone bodies utilization

Ketone bodies, comprising acetoacetate (AcAc), acetone and β-hydroxybutyrate, are generated by liver mitochondria from FAO when carbohydrate supply is stringent [117, 118]. They are transferred to extrahepatic tissues and organs, such as brain and heart as substitute fuels [119, 120]. There are metabolic analyses demonstrating that compared with healthy tissues, levels of ketone bodies in tumors are altered, where serum β-hydroxybutyrate levels are distinctly higher in PTC [121, 122], and acetone levels are significantly lower in FTC [123], suggesting ketone bodies might serve as a potential biomarker and probably TC tissues have preferred kinds of ketone bodies as the energy supply.

#### Lipid synthesis

Lipogenesis is proven to increase in cancers, which adds to the energy provision, membrane synthesis and signal transduction [124] and also obstructs the homeostasis of lipid metabolism [125]. Multiple enzymes as regulators of lipogenesis are significantly upregulated in various cancers [124]. The sterol regulator element binding proteins (SREBPs) are a family of transcription factors, modulating lipid generation including the biosynthesis of cholesterol, FA, and TG [126]. In differentiated thyroid cancer (DTC), there is evidence showing that SREBP1 level is associated with malignancy, including the size and metastasis of tumor [127]. Meanwhile, it has been demonstrated that SREBP1c activation depends on the stimulation of AKT/mTOR pathway and enhances fatty acid synthase (FASN) [128], further boosting lipogenesis. As for FASN, it has turned out to overexpress in several types of TC, including PTC [103, 129, 130] and ATC [131, 132]. Acetyl coenzyme A carboxylase (ACC) as another key enzyme in lipogenesis, catalyzes the conversion of AcCoA into malonyl-CoA and inhibits FAO [133]. It has been reported that in *BRAF*^V600E^ PTC, ACC2 expression is significantly lower compared with *BRAF*^WT^ PTC, thus downregulates lipogenesis and promotes FAO [134], indicating different metabolic tendencies in diverse genotypes of TC. Complementally, data from previous research suggest that AMPK pathway is activated in PTC, resulting in the increased pACC [135]. Aberrant stearoyl-CoA desaturase (SCD) expression is also quite common, especially in ATC [131, 136]. It has also been elucidated that DTX4 could exacerbate TC progression through upregulating SCD1 [137], which plays a decisive part in FA synthesis [138]. The increase of SCD1 in PTC is also detected [103], but deeper regulatory mechanisms are to be uncovered. Additionally, phospholipids especially phosphocholines (PCs) as an indispensable part of biological membrane, have abnormal distributions in cancers [139]. There’s little research on phospholipid synthesis in TC, but it has early been proved that in rat thyroid tumor line 1-5G, phospholipid levels are 50% lower than normal, in response to thyrotropin (TSH) stimulation and receptor upregulation [140]. Lipidomic analysis reveals the upregulated PC, phosphatidylcholine, phosphoethanolamine and sphingomyelin in PTC [141, 142] and FTC [99], sensitively distinguishing malignant TC from benign [143, 144]. Enzymatic research is expected to provide a full view of TC lipid metabolism.

#### Phospholipid degradation

Phospholipid degradation is a significant part of lipid metabolism. In TC, the remodeling of phospholipid degradation affecting tumor progression is widely investigated. Phospholipase as a crucial enzyme catalyzing phospholipid hydrolyzation [145], exhibits abnormal expressions in TC. Phospholipase C Delta 3 (PLCD3) is verified as an oncogene in TC and the PLCD3 level is closely related to the tumor progression through hippo pathway probably and can serve as an auxiliary clinical diagnostic tool or a therapeutic target [146]. Relatively, PLCD3 level is modulated by *circ_0003747*/*miR-338-3p* axis, and suppressing PLCD3 expression reduces tumor size and growth [147]. Other enzymes participating in phospholipid degradation, such as sphingomyelinase, had little research proof. However, since phospholipid metabolism categorically determines the biosynthesis of cell membrane as well as essential signal transduction involved in tumor progression, it is worth far more research.

#### Amino acid metabolism in TC

**Fig. 4:**
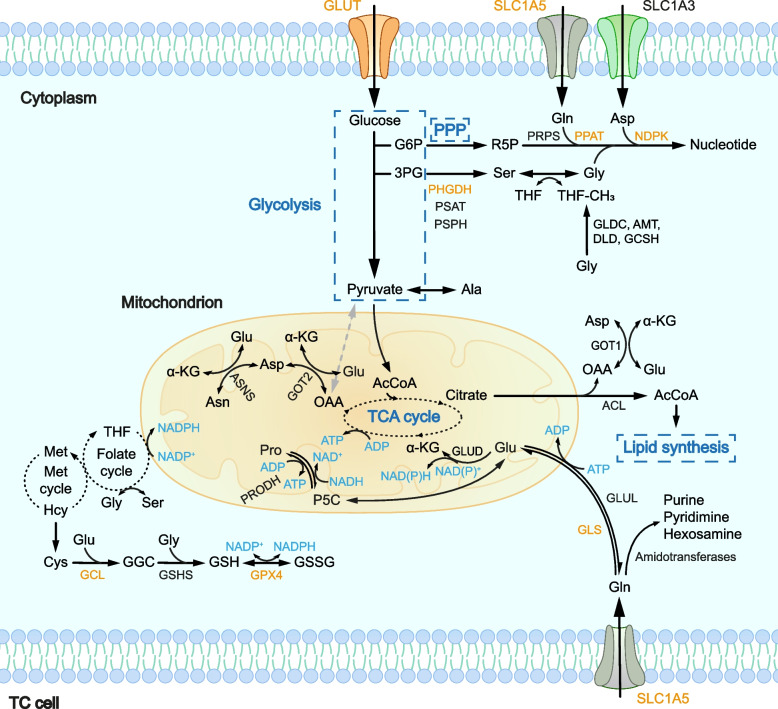
Amino acid and nucleotide metabolism in TC. Amino acids as constituents of proteins and materials of nucleotides, play an important part in linking metabolism of different substances. By regulating activities of multiple enzymes, TC cells reach a status where biosynthesis is far beyond sufficient, allowing rapid proliferation and metastasis.Abbreviations: PSAT, phosphoserine amino transferase; PSPH, phosphoserine phosphatase; THF, tetrahydrofolate; AMT, aminomethyltransferase; DLD, dihydrolipoamide dehydrogenase; GCSH, glycine cleavage system protein H; Asn, asparagine; ASNS, asparagine synthase; GLUL, glutamate-ammonia ligase; Gly, glycine; Met, methionine; Hcy, homocysteine; Cys, cysteine; GSHS, glutathione synthase; ACL, ATP citrate lyase; GOT, glutamate oxaloacetate transaminase; P5C, pyrroline 5-carboxylate; Pro, proline; PRODH, proline dehydrogenase

It can’t be overemphasized how important amino acids are in both structural and functional aspects of normal cells as well as cancer cells. As nutrients and metabolic regulators, amino acids form a unique network in accord with the altered lifestyle and requirements of cancer cells [148]. The diversified products of amino acid metabolism, such as AcCoA, pyruvate and fumaric acid, are important substrates for other processes. The speedily proliferating cancer cells demand adequate amino acids for protein synthesis, which is the primary mission of amino acids [149]. A metabolomics analysis revealed that in TC, intermediates of branched-chain amino acid (BCAA) metabolism significantly enriched. Simultaneously, the TCA cycle is activated and this activation may be associated with the entry of BCAA metabolites into the TCA cycle, thereby providing tumor cells with energy and metabolic intermediates [150]. The aberrant amino acid metabolism in TC is arousing more interest, especially glutamine, proline, serine and glycine. Other significant amino acids such as arginine, valine and leucine have few investigations on TC, but the rough metabolic framework of amino acids in TC is established. Figure 4 shows the intricate amino acid metabolic network, and the identified enzymatic changes in amino acid metabolism.

#### Glutamine

Glutamine, the most consumed amino acid in cancer cells, plays an important part in providing substrates for not only protein synthesis but also lipid synthesis [151]. As a non-essential amino acid, glutamine can be generated through endogenous synthetic pathway [152]. By glutamine catabolism, the TCA cycle is enhanced for α-KG is produced, which means glutamine serves as an energy fuel [153]. And cancer cells should utilize more glutamine to sustain their life events. SLC1A5, which is also named as ASCT2, was proven to overexpress in PTC under the regulation of lncRNA ABHD11 antisense RNA 1 (*ABHD11-AS1*), transporting quite more glutamine into TC cells [154]. The overexpression of SLC1A5 in PTC, which transports more glutamine into TC cells, is significant as glutamine not only provides the necessary building blocks for protein synthesis but also fuels the TCA cycle. This enhanced glutamine utilization is a key metabolic adaptation of TC cells to support their rapid proliferation and metabolic needs. It has also been found that SLC1A5 level, together with glutaminase 1 (GLS1) and glutamate dehydrogenase (GLUD) expressions show significant differences among diversified thyroid neoplasms, incorporating follicular adenomas, FTC and Hürthle cell neoplasms [155]. This reflects the disparity of glutamine metabolism in different types of TC. Taken all together, enzymes that raise glutamine import, including SLC1A5, GLS and GLUD, are upregulated [156], indicating the high rates of absorbing and utilizing glutamine. In addition, glutamate-cysteine ligase (GCL), converting glutamine and cysteine to glutathione, has been discovered to be upregulated in PTC-derived cell lines B-CPAP and K1, confirming glutamine’s participation in maintaining redox balance [157]. Despite glutamine metabolism in cancers is the most studied among amino acids, there are still plenty of scientific questions to be solved in TC.

#### Serine and glycine metabolism

Serine and glycine are usually discussed together, for they are linked together, providing essentials for the biosynthesis of macromolecules and maintaining redox balance [151, 158]. They are not only involved in protein biosynthesis, but also participate in one-carbon metabolism [159]. There has been ample evidence for the contributions of reprogrammed serine and glycine metabolism in cancer progression [158]. In PTC, high level of phosphoglycerate dehydrogenase (PHGDH), which catalyzes the process producing the precursor of serine as well as the first key enzyme of one-carbon metabolism, facilitates TC progression [160, 161]. Similarly, other serine/glycine metabolism-related enzymes, including phosphoserine aminotransferase, phosphoserine phosphatase, serine hydromethyl transferase (SHMT), and glycine decarboxylase differently express in various TC types covering PTC, ATC, FTC, MTC and PDTC [162]. Even more to the point, serine/glycine metabolism intensively interacts with other metabolite metabolism, which means we ought to observe the metabolic transforming wholly and exactly.

#### Proline metabolism

Proline chiefly takes part in collagen biosynthesis, which is indispensable for tumor transformation and metastasis [151]. There’s analysis supporting that proline can be considered as a biomarker assisting diagnosis and prognosis [163]. In a diagnostic study, the combination of alanine, valine, proline, phenylalanine has high sensitivity in early diagnosis for PTC [164], further verifying alteration of proline expression and its monitoring potential in PTC. Although in several types of cancer, it has been proved that enzymes related to proline metabolism express differently [151], the underlying mechanism of aberrant proline expression in TC is not clear enough, waiting for further exploration.

#### TCA cycle in TC: mitochondria-integrated metabolism

Mitochondria, long considered as the energy factory in most cells, principally undertake the process of OXPHOS, converting AcCoA to ATP through the TCA cycle [165]. As is mentioned, AcCoA can be produced by glucose, lipids and amino acids. Taking glucose as an example, after being transformed to pyruvate in the cytoplasmic matrix, the metabolite is absorbed by mitochondria and changed into AcCoA by pyruvate dehydrogenase and then enters the TCA cycle. The whole process, with the participation of oxygen, allows one molecule of glucose to generate 32 to 36 molecules of ATP, which is far more than glycolytic pathway [166]. Whereas cancer cells tend to have a partiality for glycolysis in pursuit of a more rapid energy supply [167]. In like manner, in most cancers, the ability to oxidate other substrates completely of mitochondria is weakened, hence the generation of AcCoA and the conversion of AcCoA into ATP is both decreased. This abnormality results from intricate metabolic reprogramming, which is not thoroughly investigated. But clues of abnormal mitochondria biosynthesis, fusion, fission and mitophagy are well observed in TC [168–171]. Researchers have identified a significantly elevated mitophagy associated gene, *GGCT*, through bioinformatics analysis of TCGA-ThyC dataset, with GGCT emerging as the most potent predictor. Moreover, the prognosis analysis validated the risk prediction precision of GGCT [168]. Mitochondria-eating protein (MIEAP) is a key molecule involved in non-canonical mitophagy which is independent of the PTEN-induced kinase 1 (PINK1)/Parkin pathway, and it is considered as a tumor suppressor. Mechanically, researchers have demonstrated that in TC, the absence of MIEAP expression, which indicates impaired MIEAP-mediated noncanonical mitophagy, leads to the accumulation of dysfunctional mitochondria [172]. Subsequently, they conducted in vivo experiments to validate the tumor-suppressive role of MIEAP and found that the concurrent knockout of MIEAP and autophagy-related gene 5 (ATG5) accelerated TC development and induced the oncocytic phenotype in BRAF^V600E^-positive thyroid cells [169]. Similarly, the mitochondria morphology in TC cells varied compared to normal cells. There is evidence that several “mitochondria-shaping” proteins such as mitochondrial fusion (Opa1, Mfn1 and Mfn2) and fission (Drp1 and Fis1) proteins are significantly elevated. In vitro experiments found that the blockage of Drp1 through genetic and pharmacological methods inhibited TC migration and invasion [170]. Moreover, the aberrant OXPHOS complexes are also involved in mitochondria dysfunction. Researchers have found an increased mitochondria biogenesis and OXPHOS complexes II-IV while the complex I was reduced [171]. In summary, the aberrant TC mitochondria cause oxidative damage to biomolecules, genomic instability and metabolic rewiring, which contribute to tumor progression [173]. These discoveries encourage us to dig out the underlying mechanisms even positively.

#### HIF-1α as a switch in mitochondria functioning

In the hypoxic microenvironment of cancer cells, hypoxia-inducible factor-1 (HIF-1), mostly HIF-1α, plays a pivotal role in mitochondrial abnormality as a transcriptional factor [174]. The activity of HIF-1α is regulated by oxygen levels, where normoxia promotes HIF-1α degradation and dysfunction while hypoxia ensures its binding to hypoxia response elements, augmenting the transcription of target genes [175]. Is hypoxia the cause or effect of tumors? It is not clear yet. We cannot decide whether the upregulation of HIF-1α is induced by the hypoxic milieu of the rapid growing tumor, or it is just the initial mutation of carcinogenesis. But affected by HIF-1α, the metabolism in TC is proven to change significantly. Research has found the lifted level of HIF-1α in PTC, and HIF-1α expression is related to carcinogenesis and TC progression [176]. In vivo* and vitro* experiments revealed that hypoxic stress enhances yes-associated protein (YAP) binding to HIF-1α, activating GLUT1 transcription thus promotes glycolysis in PTC [177]. Likewise, high levels of KLF5, which also binds to HIF-1α, promote PTC growth [178]. Additionally, *siHIF-1α* modulates the nuclear factor-κB (NF-κB)/PKM2 signaling pathway, influencing glycolysis [179]. And hypoxia together with estrogen are proven to affect HIF-1α expression in TC cells, providing new targets for TC treatment [180]. Also, hypoxia-induced HIF-1α/CPT1A pathway activation alters lipid metabolism, increases oxidative stress and accelerates PTC cell growth [181]. Apart from disabling aerobic oxidation, HIF-1α interferes mitochondrial biogenesis and damages mitochondrial functions as well [182].

#### Mutations of key enzymes of the TCA cycle in TC

By producing reducing equivalents in NADH and FADH_2_, mitochondria oxidate substrates through the TCA cycle, not only catabolizing bioenergy fuels, but also providing precursors for other anabolic processes [183]. As a complex and critical pathway, the TCA cycle has been reported to mutate greatly in cancers including but not limited to TC. And there’s evidence revealing that the mutations of key enzymes of the TCA cycle exist broadly in TC.

After AcCoA is produced, it participates in the loop of oxaloacetate utilization and regeneration catalyzed by a sequence of enzymes. In a gene detecting research, mutations on G367A and V71I of isocitrate dehydrogenase 1 (IDH1), which converts α-KG to 2-hydroxyglutarate are discovered in several cases of different types of TC [184], but the regulating mechanism still needs exploration. The discovery of IDH1 mutations in TC suggests a disruption in the normal TCA cycle function. These mutations may lead to the accumulation of abnormal metabolites, which can potentially affect the redox balance and energy production in TC cells, ultimately contributing to tumor progression and potentially serving as a biomarker for TC diagnosis and prognosis. Studies show mutations of succinate dehydrogenase complex (SDH) could also act as a biomarker of TC [185, 186]. Complementally, SDHD-G12S/H50R mutations are proven to promote PTEN mono-ubiquitination and downregulate autophagy in TC cells [187]. These SDH variants directly influence the survival of TC cells, probably through regulating the TCA cycle due to the function of enzyme. Fumarate hydratase also presents a novel variant in a case of PDTC [188]. Though lacking enough evidence to expose the holistic alterations in the TCA cycle, the damage of mitochondria and the TCA cycle couldn’t be overlooked for its essentiality in TC metabolic reprogramming.

#### Nucleotide metabolism in TC

Nucleotides, monomer units of DNA and RNA, join in biosynthesis, act as mediators of cell activities and also exchange and store energy [189, 190]. While in cancers, the increased nucleotide synthesis and utilization of nucleotide triphosphates are associated with cancer cells’ invasive behaviors [191]. For nucleotide synthesis, pivotal enzymes were proven to alter in TC (see Fig. 4). Levels of PPAT, the key enzyme of de novo purine nucleotide synthesis, are increased in TC tissues, enhancing PKM2 expression and activating extracellular signal-regulated kinase (ERK) and signal transducer of activation 3 (STAT3) signaling pathways [73]. Also, nucleoside diphosphate kinase (NDPK) has long been verified to be upregulated by immunohistochemical analysis in TC but recent research progress on NDPK is lacked [192–194]. And PPP, as the common pathway of glucose and nucleotide metabolism, is stated before. Other key regulators such as phosphoribosyl pyrophosphate synthetase and nucleotide catabolism-related enzymes have little evidence, but in consideration of the high speed of cell division, we can speculate about the great change of nucleotide metabolism and the critical regulators require in-depth study.

## Metabolism in TC influenced by physiological variations

There has been abundant research demonstrating the close linking of physiological variation and cancer progression, and in TC, metabolism is also influenced by the alterations of physiological status. An increasing number of studies have suggested an association between obesity and the development of TC. An Australian study estimated that nearly one in five future TC cases could be attributed to current overweight or obesity [195]. The statistic is 11.4% to 16.2% between 1995–2015 in the United States [196]. However, the precise mechanisms underlying their crosstalk remain unclear. Current research indicates that fat tissue secreted adipokines such as leptin and adiponectin may be implicated in the link between overweight status and TC metabolism [197]. Acrp30 (also known as adiponectin) and leptin are also involved in TC progression. Researchers have shown that Acrp30 inhibits the proliferation of TC cells [198]. Agonists targeting the receptors of Acrp30 not only inhibit the proliferation of thyroid cancer cells but also suppress intracellular amino acid metabolism and glucose metabolism [199]. Emerging evidence has established that obesity is a significant risk factor that can influence the metastasis of TC through multiple pathways, including dysregulation of adipokine expression. These findings underscore the urgency of health management for individuals with obesity and the necessity of preventive strategies for TC.

TC is a typical tumor whose incidence is closely associated with gender and age, characterized by a higher prevalence in females, especially those of childbearing age. This gender disparity may be related to multiple factors, including genetics, hormones, and health awareness. The X-chromosome inactivation (XCI)-related gene, *XIST*, is responsible for XCI. Under physiological conditions, this process occurs only in female cells to balance the dosage between the two X chromosomes [200]. Interestingly, XIST is significantly upregulated in thyroid cancer cells. It can activate the hepatocyte growth factor receptor (MET)/PI3K/AKT signaling pathway [201] and inhibit the expression of Claudin-1 [202], thereby promoting the proliferation, migration, and invasion of TC cells. Intriguingly, XIST can also enhance HIF-1α expression via the XIST/*miR-93*/HIF-1α pathway, thereby promoting glycolysis in TC [203]. Therefore, the high expression of XIST in females may at least partially explain the gender predisposition in the incidence of TC. Additionally, the role of estrogen cannot be overlooked. The perimenopausal period (45–55 years) is characterized by significant fluctuations in estrogen levels among women. Prior to menopause, estrogen levels may transiently increase [204]. Studies have demonstrated that estrogen exerts regulatory effects on gene expression. Specifically, it enhances the recruitment of DNMT3B to the FAM111B enhancer. The methylation of FAM11B mediated by DNMT3B promotes glycolysis in PTC cells. Additionally, the expression of estrogen can also account for the higher incidence of TC in women of childbearing age. Elderly men often suffer from metabolic syndrome, including type 2 diabetes and hyperlipidemia. These aberrant metabolic patterns may provide substrates for glycolysis and lipid metabolism in TC [205]. Additionally, hyperglycemia may impair mitochondrial function, thereby affecting the expression of oxidative phosphorylation enzymes and reducing substrate utilization, thus altering the glucose metabolic patterns in TC [206]. Research on metabolic alterations in elderly male thyroid cancer patients are limited, and further mechanistic studies focusing on the elderly population are needed to elucidate this topic.

## Novel regulators in TC metabolism

### Vitamin-related regulators

Recent evidence shows vitamins, including vitamin D, vitamin C and so on, play a part in TC development. These vitamins either serve as metabolic regulators or directly influence the overall state of the body, deciding on the response capability to cancer cells.

Vitamin D as a fat-soluble secosteroid, mainly participating in calcium metabolism [207], has to do with the carcinogenesis and progress of TC. In a meta-analysis, vitamin D deficiency is a risk factor for TC [208]. Study has shown that rs12785878 minor allele of DHCR7, converting 7-dehydrocholesterol (7-DHC) to cholesterol in vitamin D synthesis, is relevant to the susceptibility to TC [209]. This implies that maintaining adequate vitamin D levels may be a protective factor against TC development. Although investigations on the downstream pathways of vitamin D in TC are limited, we can anchor our hope in its anticancer effects and multidimensional regulatory roles in immune status, TME and cancer cell metabolism [210, 211].

Vitamin C, also named as ascorbic acid, is one of the exogenous nutrients that have to be obtained from diets [212]. Recently vitamin C has become a promising anti-cancer agent by inducing pro-oxidative cytotoxicity in cancer cells, enhancing the levels of anti-cancer regulators, blocking signal transduction and improving immune response [213]. In TC, high-dose of vitamin C is proven to kill cancer cells through ROS-dependent inhibition of MAPK/ERK and PI3K/AKT pathways [214]. And interference of redox balance is observed in Vitamin C-treated PTC, bringing about apoptosis and necrosis [215]. Additionally, Vitamin C augments the anti-cancer effect of PLX4032, which is a selective inhibitor of the *BRAF*^V600E^ kinase [216]. In ATC, Vitamin C induces ferroptosis, which is also associated with ROS [217], further demonstrating the pro-oxidant effect of vitamin C. Furthermore, vitamin C decreases HIF-1α level in TC cells, thus downregulating GLUT1 expression [218].

Other vitamins, for instance, vitamin A and vitamin E, could be in connection with TC metabolism as well. Retinoic acid as the derivative of vitamin A, promotes the activity of iodine uptake, and sodium-iodine symporter (NIS) and retinoids widely regulate gene expression by interacting with nuclear receptors [219]. Since NIS plays a pivotal role in hormone synthesis [220], we can estimate the importance of vitamin A. Overexpression of cellular retinoic acid-binding protein 2 (CRABP2) adds to the epithelial–mesenchymal transition and adverse prognosis of TC [221]. The role of vitamin E in TC treatment mainly concentrates on its protective function of salivary glands from radioiodine therapy [222–224], while the effect of vitamin E in cancer prevention and therapy is broadly researched [225–227]. Thus, the therapeutic potential of vitamin E in TC treatment deserves more attention.

### Oxidative stress regulating TC metabolism

Oxidative stress is a state where typically ROS generation and antioxidation lose balance and this imbalance is extensively involved in cancer advancement [228]. The role of ROS varies with its concentration, which means, ROS acts as a regulator at low levels and a destroyer when its concentrations are high [229]. It is widely proved that the increase of ROS promotes various types of cancers by activating specific signal pathways, damaging genetic stability and influencing cell survival and death [230]. Produced in cells, ROS can be induced by both endogenous and exogenous factors via varieties of approaches, and high levels of ROS are closely related to malignancies [231].

In TC, it was demonstrated that high-mobility group box 1 (HMGB1) mediates autophagy, which promotes NIS degradation via ROS/AMPK/mTOR pathway [232], revealing the cell death-modulating role of ROS. As one of the excessive metabolites, ROS is certified as a driver of oncogenesis by triggering loss of whole chromosomes in the metabolically relatively quiescent cell lines of FTC [233], suggesting that ROS may have an effect on metabolic manipulation. Another research found the mitochondrial DNA (mtDNA) mutation G3842A injures mitochondrial complex I, influences oxygen consumption and ATP production and elevates ROS levels, thus activating ERK1/2 signaling and facilitating carcinogenesis [234]. An consensus clustering analysis found the high levels of NDUFB3 adds to the mitochondrial ROS (mitoROS) and supervision of mitoROS regulators may appear clinical value [235]. What’s worth mentioning is that ROS is directly corelated to ferroptosis, the type of cell death following iron-dependent lipid peroxidation [236]. And upregulation of glutathione peroxidase 4 (GPX4) is observed in TC, obstructing ferroptosis and promoting oncogenesis [237]. Inhibition of GPX4 rehabilitates ferroptosis in polymorphic TC cells, which results from ROS accumulation and GSH depletion [238].

Overall, the impact of oxidative stress is more than merely on metabolic reprogramming or cell survival. On the one hand, oxidative stress is the effect of metabolic alterations. On the other hand, it triggers the reconstruction of cancer cells and tumor microenvironment in return. And the mechanism of the subverted redox balance and oxidative stress is under exploration.

### Other potential regulators

As an endocrine organ, except for producing hormones, the metabolism of thyroid itself is also regulated by hormones. There’s evidence supporting that high triiodothyronine, thyroxin and thyroid hormone (TH) levels are pro-cancer factors [239]. Moreover, TSH in the TME has an effect on TC with high TSHR expressions, boosting tumor growth and metastasis [240]. With plenty of research focusing on TH and TSH in postoperative therapy, the original hormone levels are worthy of focus. Besides, metabolic reprogramming and epigenetic modifications interact with each other, enabling further tumor progression [241]. *BRAF* mutation is common in TC and leads to considerable metabolic rewiring, the epigenetic modifications of *PTEN* and other genes are widely observed as well [242]. Iodide levels also regulate HIF-1 and vascular endothelial growth factor (VEGF) expressions, influencing cancer cell metabolism and angiogenesis [243]. With more novel regulators being detected, their clinical applications could benefit more patients.

See Table 1 for a comprehensive summary of metabolic regulators in TC.

**Table 1:** Key metabolic regulators associated with TC progression

Regulator	Metabolism regulated	Upregulated or downregulated in malignant TC	Effects in TC progression	Participation pathway/ downstream molecules	Mechanism	Reference
lncRNA *NBR2*	Glucose and lipid metabolism	Downregulated	Negative	AMPK signaling	Decreasing GLUT1 expression, upregulating AMPK and ACC phosphorylation, and thus regulating glucose and lipid metabolism and inhibiting TC growth	[45]
FTO	Glucose metabolism	Downregulated	Negative	APOE/IL-6/JAK2/STAT3 pathway	Downregulating APOE mRNA m6A modification and inhibiting PTC glycolysis via IL-6/JAK2/STAT3 pathway	[46]
PTEN	Glucose metabolism	Downregulated	Negative	PI3K/AKT pathway	Controlling GLUT1 expression and relocation, consequently determining the glucose uptake of TC	[47]
SIRT6	Glucose metabolism	Upregulated	Positive	HIF-1α	Upregulating GLUT1, HK2 and GADPH expressions, increasing glucose uptake, lactate, ROS, and ATP production	[48, 49]
Afamin	Glucose metabolism	Upregulated	Positive	AKT/mTOR pathway	Upregulating glucose metabolism in TC by increasing expressions of key enzymes including GLUT1, HK2 and PARP1	[50]
*BRAF*^V600E^	Glucose, lipid and iodine metabolism	Upregulated	Positive	GLUT1, PKM2, SLC5A8, ACC2	Increasing GLUT1 and PKM2 levels, reducing expressions of SLC5A8, ACC2 and key genes associated with iodine metabolism, augmenting TC aggression	[51, 72, 107, 134]
Six1	Glucose metabolism	Upregulated	Positive	GLUT3	Regulating genes including GLUT3 and increasing glycolysis as well as lactate and ATP production, thus boosting PTC invasion	[53]
E2	Glucose metabolism	Upregulated	Positive	E2/DNMT3B/FAM111B pathway	Inducing DNMT3B-mediated FAM111B methylation, causing upregulation of glycolytic gene PGK1 and accelerated PTC progression	[54]
*circRAD18*	Glucose metabolism	Upregulated	Positive	*circRAD18*/*miR-516b*/ PDK1 axis	Increasing glucose uptake and lactate production by regulating *miR-516b*/PDK1 axis	[55]
ANP32E	Glucose metabolism	Upregulated	Positive	AKT/mTOR/HK2 pathway	Promoting TC progression through AKT/mTOR/HK2-induced glycolysis upregulation	[67]
*circNRIP1*	Glucose metabolism	Upregulated	Positive	*miR-541-5p*, *miR-3064-5p*/PKM2 axis	Sponging *miR-541-5p* and *miR-3064-5p* and together promoting PTC progression by upregulating PKM2 and facilitating PTC progression	[69]
AMPK	Glucose and lipid metabolism	Upregulated	Positive	PKM2, β-catenin, ACC	Recruiting PKM2 and β-catenin, thus promoting transcription of migration-related genes; increasing ACC phosphorylation initiating carcinogenesis	[70, 135]
*miR-148a*, *miR-326*	Glucose metabolism	Downregulated	Negative	PKM2	Downregulation of *miR-148a* and *miR-326* leads to the overexpression of PKM2, resulting in thyroid carcinogenesis	[71]
PPAT	Glucose and nucleotide metabolism	Upregulated	Positive	PKM2, ERK, STAT3	Activating ERK an STAT3 pathways and lifting PKM2 expression, thus promoting TC advancement	[73]
SLC27A6	Lipid metabolism	Upregulated	Positive	c-MYC	Enhancing PTC progression by upregulating c-MYC	[106]
lncRNA *SOCS2-AS1*	Lipid metabolism	Upregulated	Positive	p53	Accelerating the degradation of p53, improving PTC cell proliferation and FAO rate	[116]
Pyruvate carboxylase	Lipid metabolism	Upregulated	Positive	AKT/mTOR/SREBP1c /FASN axis	Activating AKT/mTOR/SREBP1c axis, thus inducing lipogenesis by FASN and promoting PTC invasion	[128]
DTX4	Lipid metabolism	Upregulated	Positive	SCD1	Regulating lipid metabolism via SCD1, thus exacerbating PTC status	[137]
PLCD3	/	Upregulated	Positive	Hippo pathway	Benefiting tumorigenesis and progression of TC, and inhibiting TC cell apoptosis by activating hippo pathway	[146]
*circ_0003747*	/	Upregulated	Positive	*miR-338-3p*/PLCD3 axis	Downregulating *miR-338-3p* and relieving its suppression on PLCD3, thus facilitating TC progression	[147]
lncRNA *ABHD11-AS1*	Amino acid metabolism	Upregulated	Positive	*miR-199a-5p*/SLC1A5 axis	Stimulating TC progression by activating *miR-199a-5p*/SLC1A5 axis, conveying adequate glutamine supply	[154]
GCL, GLS1	Amino acid metabolism	Upregulated	Positive	GSH/GSSG ratio, cysteine/cystine ratio, metabolites in TCA cycle	Regulating redox status and energy production in PTC by adjusting various metabolic pathways, thus boosting TC progression	[157]
YAP	Glucose and iodine metabolism	Upregulated	Positive	HIF-1α, GLUT1, NIS	The enhanced binding of YAP to HIF-1α promotes GLUT expression and reduces NIS levels, reprogramming glucose and iodine metabolism in PTC	[177]
KLF5	/	Upregulated	Positive	HIF-1α	Binding to HIF-1α, promoting PTC development	[178]
HIF-1α	Lipid and mitochondria metabolism	Upregulated	Positive	CPT1A	Reprogramming lipid metabolism, increasing oxidative stress, preventing mitochondria biosynthesis and functions, thus accelerating PTC cell growth	[114]
SDHD-G12S/H50R	Mitochondria metabolism	Upregulated	Positive	PTEN	Promoting PTEN mono-ubiquitination and downregulating autophagy in TC cells	[187]
DHCR7 rs12785878 minor allele	Lipid metabolism	Upregulated	Positive	Vitamin D	Increasing the risk of TC probably by regulating circulating vitamin D levels	[209]
Vitamin C	Glucose and mitochondria metabolism	Downregulated	Negative	MAPK/ERK and PI3K/AKT pathways, ROS, HIF-1α, GLUT1	Inhibiting MAPK/ERK and PI3K/AKT pathways through ROS, inducing PTC cell apoptosis and necrosis and ATC cell ferroptosis, decreasing HIF-1α levels and thus reducing GLUT1 expression	[214]
Retinoic acid	Iodine metabolism	Downregulated	Negative	NIS	Increasing iodine uptake and NIS activity thus impeding TC advancement	[219]
CRABP2	/	Upregulated	Positive	Integrin/FAK/AKT pathway	Promoting TC recurrence and invasion through integrin/FAK/AKT pathway	[221]
HMGB1	Iodine metabolism	Upregulated	Positive	ROS/AMPK/mTOR pathway	Mediating autophagy-induced NIS degradation via ROS/AMPK/mTOR pathway, thus influencing the resistance to radiotherapy in TC	[232]
mtDNA G3842A	Mitochondria metabolism	Upregulated	Positive	Mitochondria complex I, ERK1/2 signaling	Impairing mitochondria function by injuring complex I, and activating ROS-induced ERK1/2 signaling, thus promoting TC tumorigenesis	[234]
NDUFB3	Mitochondria metabolism	Downregulated	Negative	mitoROS	Lifting mitoROS levels, ATP production and complex I activity in TC cells, thus preventing TC growth	[235]
GPX4	Lipid metabolism	Upregulated	Positive	mTOR pathway	Regulating mTOR pathway and promoting TC carcinogenesis by inhibiting ferroptosis as well as regulating ROS accumulation and GSH metabolism	[237, 238, 244]
Iodide	Iodide and mitochondria metabolism	downregulated	negative	VEGF, NIS, ROS	Lack of iodide increases ROS levels, thus stimulating TC angiogenesis via HIF-1-activated VEGF, while excess iodide suppresses NIS expression and displays the opposite effects	[243]

## TC detection and therapy targeting metabolic reprogramming

### Detecting methods based on redirected metabolism

The traditional detecting strategy for TC is physical examination and imaging modality, and biopsy provides evidence for subtype diagnosis [245]. While ultrasound is the preferred choice for suspected thyroid nodules, fine-needle aspiration offers proof for benign or malignant diagnosis [246]. Recent progress in TC metabolism research has boosted molecular assessment based on metabolic reprogramming (see Table 2).

**Table 2:** Detections based on the reprogrammed metabolism of TC

Detection strategy	Principle	Detection index	Reference
^18^F-FDG PET or PET/CT	^18^F-FDG can enter glycolysis	Fluorescence intensity	[247–249]
^18^F-FGln PET/CT	Gln uptake increases significantly in TC	Fluorescence intensity	[250]
^18^F-TFB	Mimicking iodine transport via NIS in NIS-expressing tissues	Fluorescence intensity	[251]
TiO2@COF-based SPME combined with UHPLC-MS/MS	Recognizing PLs in TC serum	Serum PL concentration	[252]
DESI-MSI	Revealing lipid distribution	Compound abundance	[253]
Raman microscopy	Analyzing TC cell locations and biochemical characters	Subcellular distinctions in the Raman images	[254, 255]
HPLC-TSQ-MS	Profiling serum amino acid metabolites of TC patients	Abundance of amino acids	[256]

Among metabolism-based detections, ^18^F-fluorodeoxyglucose (^18^F-FDG) position emission tomography (PET) and PET/computed tomography (PET/CT) have been put into use for a long period in various types of cancers including TC [247]. FDG can pass the cell membrane via GLUT and enter glycolytic pathway. In the stable form of FDG-6-phosphatate (FDG-6-P), the fluorescence intensity is detected, reflecting the activity of GLUT of the tissue under test [248]. It can assist in early diagnosis, staging, and monitoring treatment response in TC patients with its non-invasive advantage. A new clinical trial compared the detecting value of ^68^ Ga-prostate-specific membrane antigen 11 (PSMA-11) PET/CT and ^18^F-FDG PET, finding both of them can inspect TC metastasis but ^18^F-FDG PET is more accurate [249]. Moreover, dynamic imaging of ^18^F-(2S,4R)4-fluoroglutamine (^18^F-FGln) PET/CT can be a complement of ^18^F-FDG PET/CT in TC detection [250]. Recent research on ^18^F-Tetrafluoroborate (^18^F-TFB) and its analogs for NIS and ^68^ Ga-tetraazacyclododecane tetraacetic acid-DPhe1-Tyr3-octreotate (^68^ Ga-DOTATATE) PET/CT has been promising equally well [251]. And for lipid detection, TiO_2_ and covalent organic frameworks (COFs) are able to adsorb PCs and lysophosphatidylcholines and thus TiO_2_@COF-based solid-phase microextraction (SPME) combined with ultra-high-performance liquid chromatography-tandem mass spectrometry (UHPLC-MS/MS) was invented, providing a reliable strategy for TC early diagnosis [252]. Desorption electrospray ionization mass spectrometry (DESI-MS), which mainly shows lipid distribution, was applied in TC metastasis imaging displayed high signals of ceramides and glycerophosphoinisitols in TC metastasis [253]. In Raman microscopic images, FTC cells incorporate more lipid constituents and fewer cytochrome components than normal cells [254]. Raman spectroscopy also shows high sensitivities, specificities and accuracies in differentiating benign cells and malignant TC subtypes by monitoring nucleic acids, lipids, carbohydrates and protein [255]. Serum metabolomics analysis through high-performance liquid chromatography-triple stage quadrupole-mass spectrometry (HPLC-TSQ-MS) has found the amino acid distinctions between PTC with or without Hashimoto's thyroiditis (HT) and healthy samples, which not only distinguishes PTC patients from normal people, but also identifies patients with HT [256]. This serum-plasma metabolite detection can serve as an auxiliary tool.

With the progress of science and technology, new diagnostic means will constantly come into being, rendering personalized medicine to each demander. Consequently, only by combining multifaceted detecting strategies can we avail the existing tools to the utmost extent and benefit more patients.

### Metabolic targets in TC therapy

**Fig. 5:**
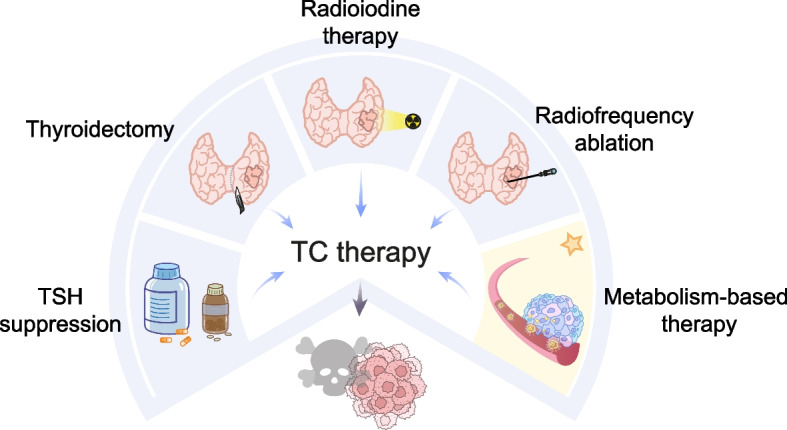
Therapeutic strategies of TC

While thyroidectomy, radioiodine therapy and TSH suppression make up the mainstream of TC treatment, targeted therapies restraining pathways involved with MAPK, PI3K/AKT/mTOR or VEGF are on the rise [257–259]. Figure 5 displays the classical and novel treatment strategies of TC. Featured with significantly reprogrammed metabolism, TC treatment aiming at metabolic targets has become a new trend and we summarized the metabolism-targeting strategies of different categories in Table 3, hoping to give research and medication guidance.

**Table 3:** Metabolic targets and their potency in blocking TC

**Drug/substance**	**Metabolic target**	**Effect**	**Reference**
*circ_100395*	PI3K/AKT/mTOR pathway	Inhibiting glucose uptake and lactate production	[260]
*sh-circPUM1*	*miR-21-5p*/MAPK1 axis	Downregulating glycolysis	[261]
Encapsulated *miR-143* agomir	HK2		[262]
STAT3	IL-6/gp130/JAK signaling		[263]
TRIM26	PI3K/AKT pathway		[264]
Metformin	LDHA, PKM2, IDH1		[265–267]
Canagliflozin	DPP-IV, SGLT2		[268, 269]
Imatinib combined with vemurafenib	PDGFR, BRAF, HK2		[270]
3-bromopyruvate	HK2		[271]
Sorafenib, cabozantinib	NIS, GLUT		[272]
*shRNA-AKR1C3-1/2*	ERK signaling		[273]
*PTCSC3*	PGK1		[274]
Pt@TAT/sPEG	LDH		[275]
6-aminonicotinamide, oxythiamine	G6PD, transketolase	Inhibiting PPP and inducing ROS-mediated apoptosis	[94]
6PGD siRNAs	6PGD	Interrupting 6PGD-induced doxorubicin resistance in ATC	[95]
Acriflavine, syrosingopine, AZD3965	MCT4	Preventing lactate export and glycolysis	[276]
JAZF1	TAK1/NF-κB pathway	Influencing cell cycle, inhibiting proliferation and causing apoptosis	[277]
CP-91,149	Glycogen phosphorylase	Decreasing NADPH levels, inducing ROS accumulation and apoptosis	[278]
CP-91,110,049		Suppressing ATC growth in vivo	
Tunicamycin	GLUT1	Reducing glucose uptake, assisting radioiodine therapy	[279]
2-DG	/	Enhancing the therapeutic effect of doxorubicin and sorafenib	[280]
Ceramide	Caspase-3-dependent pathway	Influencing the antitumor effect of camptothecin and doxorubicin on FTC	[281]
shRNA-pyruvate carboxylase	AKT/mTOR/SPEBP1c/FASN pathway	downregulating lipogenesis, thus obstructing TC progression	[128]
METTL16	SCD1	Activating lipid metabolism	[282]
AdipoRon	Adiponectin receptor	Inducing TC cell autophagy	[199]
Exosome-loaded *si-SCD1*	SCD1	Causing ROS accumulation	[138]
RSL3	GPX4	Inducing ferroptosis of TC cells	[244]
*si-circKIF4A*	*circKIF4A*/*miR-1231*/GPX4 axis	Increasing TC cell ferroptosis	[283]
SIRT4	GLUD	Inhibiting the metabolism of glutamate and glutamine	[284]
*sh-STAG2*	SLC1A5, GLS1, GLS2	Limiting glutamine intake	[285]
DON	GLS	Inhibiting the metabolism of multiple amino acids, influencing cell cycle, promoting apoptosis and impeding TC progression	[156]
Vemurafenib combined with PIM1i	BRAF, PIM1		[286]
SHIN1	SHMT	Suppressing one-carbon metabolism and causing purine depletion	[287]
DS18561882	MTHDF2		
BPTES combined with CBR-5884	GLS1, PHGDH	Inhibiting glutaminolysis and one-carbon metabolism, elevating the chemotherapy efficacy of lenvatinib and sorafenib	[161]
Capsaicin	OCT4A, TRPV1-Ca^2+^/cAMP /PKA/CREB pathway	Triggering autophagy and apoptosis	[288–291]
Piperlongumine	PI3K-AKT pathway		[292]
Disulfiram/Cu	MAPK/ERK and PI3K/AKT pathways	Sensitizing BRAF-mutant TC cells to PLX4032 by relieving ROS-dependent feedback activation	[293]
SB-204990, NDI-091143	ACLY	Raising the cytotoxicity of sorafenib	[294]
Monensin	AMPK/mTOR pathway	Inducing oxidative stress	[295]
Au–Ag@Polydopamine nanoparticle	Dihydroorotate dehydrogenase	Damaging mitochondrial function	[296]
Alantolactone	ROS-mediated mitochondria-caspase pathways	Leading to apoptosis and pyroptosis	[297]
Curcumin	SDH, ROS	Inducing mitophagy	[298]

With traditional therapies such as ^131^I therapy, surgery treatment and TSH suppression drugs developing for a long period, metabolism-based therapy has progressed much. And comprehensive treatment is becoming a trend.

### Targeting glucose metabolism

Since glucose metabolism is the most researched field in cancer metabolism, anticancer drugs blocking glucose metabolism-related enzymes and regulators are increasingly promising [299].

Primarily, inhibition of TC glycolysis exhibits a strong antitumor effect. It has been demonstrated that *circ_100395* plays a role in reducing glucose intake and lactate production by inhibiting PI3K/AKT/mTOR pathway, obstructing TC cells’ survival and metastasis [260]. And knockdown of *circPUM1* inhibits glycolysis through *miR-21-5p*/MAPK1 axis in PTC [261]. Encapsulated *miR-143* agomir is proven to target HK2 in PDTC, which decreases glycolysis and presents therapeutic value [262]. Except for non-coding RNAs, STAT3 and tripartite motif 26 (TRIM26) are also negative regulators of glycolysis in PTC [263, 264]. While hyperinsulinemia increases TC malignancy, metformin, an oral hypoglycemic drug, is verified to impede glycolysis and promote TCA cycle via downregulating LDHA and PKM2 and enhancing IDH1 expression [265, 266]. Similar to metformin, artemisinin shows its antitumor potential by decreasing HIF-1α, which suppresses glycolysis in TC [203]. Additionally, dipeptidyl peptidase-IV (DPP-IV) and sodium-glucose cotransporter 2 (SGLT2) inhibitors such as canagliflozin suppresses glycolysis and influences multiple pathways [268, 269]. Imatinib and vemurafenib block platelet-derived growth factor receptor (PDGFR) and BRAF respectively, and drug combination has a positive impact on TC elimination through inactivating HK2 [270]. 3-bromopyruvate also targeting HK2 is testified to ameliorate survival of ATC [271]. Both sorafenib and cabozantinib were confirmed to induce NIS and inhibit GLUT expression [272]. By inactivating ERK signaling, silencing of aldo–keto reductase family 1 member C3 (AKR1C3) is reported to inhibit autophagy-dependent glycolysis in TC [273]. In addition, inhibition of the overexpressed phosphoglycerate kinase 1 (PGK1) via lncRNA papillary thyroid cancer susceptibility candidate 3 (*PTCSC3*) suppresses TC progression significantly [274]. Moreover, G6PD and transketolase inhibitors, targeting PPP, were verified to induce ROS-mediated apoptosis in TC cells [94]. MCT4 inhibitors acriflavine, syrosingopine, and AZD3965, which prevent lactate export, suppress glycolysis and restrain ATC growth [276]. Juxtaposed with another zinc finger gene 1 (JAZF1), engaged in gluconeogenesis and lipid metabolism, hinders PTC progression by inhibiting TAK1/NF-κB pathway [277]. Similarly, researchers have developed a platinum nanocluster (Pt@TAT/sPEG) with pH responsiveness and nuclear targeting properties to simultaneously target lactate dehydrogenase and exacerbate DNA damage, thereby promoting the death of TC cells [275]. Meanwhile, suppression of glycogen phosphorylase induces apoptosis in ATC cells, which means glycogen metabolism provides new targets for TC treatment [278].

Targeting metabolic process can also serve as a collaborative element of other therapeutic strategies. For instance, tunicamycin downregulates GLUT1 and less ^18^F-FDG absorption is observed in ATC as well as recovering iodide-handling gene expression and radioiodine avidity, promoting more valid radioiodine therapy [279]. 2-DG can augment the PTC cells’ chemotherapy responsiveness to doxorubicin and sorafenib by diminishing dose-dependency in cell vitality, intracellular ATP consumption, and extracellular lactate production [280].

### Targeting lipid metabolism

The anticancer research on lipid metabolism mainly focused on impeding lipid synthesis and boosting ferroptosis mediated by lipid peroxidation, and some lipids themselves were proven to suppress tumor progression. It’s demonstrated that the de novo synthesis of ceramide, the important phospholipid component of ECM, enhances the effect of camptothecin and doxorubicin on FTC therapy, and inhibiting glucosylceramide synthase augments the antitumor effects even better [281]. Targeting at lipogenesis, pyruvate carboxylase is proven to activate AKT/mTOR pathway, thus upregulating SREBP1c and FASN-induced lipogenesis in TC cells, which promotes anticancer effects [128]. Analogously, methyltransferase-like protein 16 (METTL16) inhibits PTC progression by activating SCD1, which increases FA synthesis [282]. While exosome-loaded *si-SCD1* accelerates ATC cell death by ROS accumulation [138]. This contradiction suggests different types of TC may have diverse metabolic statuses, which needs validation by contrast experiment. It also implies either lack or excess of intracellular FA can cause cytotoxicity. GPX4 inhibited by RSL3 is reported to be responsible for the upregulation of TC cell ferroptosis [244]. Mediating ferroptosis in TC, *circKIF4A* also acts as a potential target [283]. As for anticancer lipids, sphingosine 1-phosphate decreases ATC migration, while activation of estrogen receptor α and β restrains PTC progression [300]. Additionally, AdipoRon is a novel adiponectin receptor agonist that inhibits glucose and amino acid metabolism in TC cells, induces autophagy, and thereby exerts antitumor effects [199]. And α-lipoic acid suppresses PTC and FTC development, which can supplement other therapies [301].

### Targeting amino acid metabolism

With the critical role in cancer cell metabolism, redox balance and epigenetic modifications, amino acids particularly glutamine, have been promising metabolic targets [302]. Sirtuin 4 (SIRT4) is certified to impede the migration and invasion of PTC cells by blocking glutamine metabolism [284]. In *BRAF*^V600E^ mutants of TC cells, stromal antigen 2 (STAG2) knockdown decreases glutamine intake and utilization by downregulating the downstream molecules (SLC1A5, GLS1 and GLS2) of ERK/AKT/GSK3β/c-MYC axis and promotes TC cells autophagy and apoptosis [285]. Investigators have demonstrated that the combination of vemurafenib and a proviral integration site for Moloney murine leukemia virus 1 (PIM1) kinase inhibitor, an oncogenic kinase implicated in cancer progression, significantly enhances the suppression of TC cell proliferation compared to vemurafenib monotherapy. This synergistic effect is attributed to the inhibition of critical amino acids essential for cancer cell metabolism, including phenylalanine, glutamate, proline, and methylated lysine, as well as the depletion of nearly all nucleotides required for DNA synthesis and repair [286]. Moreover, 6-diazo-5-oxo-l-norleucine (DON) not only suppresses GLS activity, but also diminishes proline, alanine, aspartate, asparagine, and glycine in TC cells, which blocks TC migration, invasion, and cell cycle [156]. Furthermore, serine hydroxymethyltransferase inhibitor 1 (SHIN1) as an inhibitor of SHMT and DS18561882 as an MTHFD2 inhibitor, show great anticancer effect by interfering one-carbon metabolism in ATC [287]. There’s also research demonstrating that the co-inhibition of glutamine and one-carbon metabolism improves the curative effect of chemotherapy in ATC, which adds hope to a compositive metabolic therapeutic strategy [161].

### Targeting mitochondria

Mitochondria as the metabolic intersection in cancer cells, provide multifarious therapeutic targets. It has been broadly stated that mitochondrial DNA mutations, mitophagy, mitochondrial genesis and mitochondria-mediated apoptosis play a part in TC progression [303]. Research reveals that autophagy determines mitochondrial respiration and the levels of FAO and glycolysis, which affects vemurafenib resistance in *BRAF*^V600E^ TC treatment [304]. Thus, control of autophagy may become a new direction. Research has shown that capsaicin activated by transient receptor potential vanilloid 1 (TRPV1) can induce calcium influx and octamer-binding transcription factor 4A (OCT4A) degradation, leading to autophagy and losing of stemness in ATC cells [288, 289]. In addition, capsaicin is testified to redifferentiate ATC cells by triggering TRPV1-Ca^2+^/cAMP/PKA/CREB cascade pathway, which provides new strategy assisting radioiodine therapy [290]. And capsaicin loaded in Au@mesoporous silica nanoparticles (Cap-Au@MSNs) has strong cytotoxicity in FTC and PTC cell lines by triggering apoptosis [291]. As an aside, piperlongumine has an effect on restraining FTC cell growth and cell cycle, inducing apoptosis and autophagy by PI3K/AKT pathway [292]. And disulfiram/Cu sensitizes *BRAF*^V600E^ mutants’ response to BRAF kinase inhibitors by relieving ROS-dependent feedback activation of MAPK/ERK and PI3K/AKT pathways [293]. Targeting mitochondrial enzymes is also promising. For instance, controlling ATP-citrate lyase (ACLY) by SB-204990 and NDI-091143 has antitumor effects and synergistically enhances the cytotoxicity of sorafenib in ATC and FTC [294].

Impeding mitochondrial function is also prospective. For example, investigation reveals the alternative anti-ATC ability of monensin, which gets in the way of mitochondria functioning, inducing oxidative stress and activating AMPK/mTOR pathway [295]. In a nanotherapeutic research, Au–Ag@Polydopamine nanoparticles can be absorbed by PTC cells and accumulate in the mitochondria, abidingly injuring mitochondrial function and expression of dihydroorotate dehydrogenase [296]. Alantolactone extracted from Inula helenium L has anti-ATC impact via ROS-mediated mitochondria-caspase activation, which induces apoptosis and pyroptosis [297]. Moreover, curcumin is testified to induce mitophagy by its mitochondria-targeting peculiarity, which triggers the overactivity of SDH and superabundant ROS generation [298]. Additionally, metformin not only influences glucose metabolism in TC cells, but also suppresses mitochondrial respiration, which adds to its antitumor effect [267].

## Conclusions

Puzzles of how this interlocking metabolic remoulding takes shape still remain. For example, in cancer cells, besides FAO for ATP production, the accumulated FAs can give rise to lipotoxicity [305]. And what is the primitive alteration of malignant cells? In PTC, research has discovered the loss of pyruvate dehydrogenase phosphatase regulatory (PDPR) increases phosphorylation of pyruvate dehydrogenase and influences glucose metabolism. The subsequent cohort study revealed the role of *PDPR* mutation in PTC predisposing [306]. Study in lung cancer found that tumor-derived exosomes can reprogram glycolysis of the metastatic site in advance, thus inducing M2 polarization of macrophages [307]. In breast cancer and colorectal cancer, there are similar findings that exosomes adds to the immunosuppressive metastatic microenvironment and facilitates tumor cell spreading [308]. This finding suggests that metabolism rewiring occurs in the pre-metastasis niche ahead of the migration of cancer cells, in other words, metabolic reprogramming is one of the preparations of tumor metastasis. However, in TC, such evidence is lacking, so more research has to be advocated to provide a more comprehensive view of TC metastasis. The entire metabolic rewiring depicts an indispensable portion of the whole picture of cancer mutations. The glucose metabolism as the most researched in TC, is based on Warburg effect and has extended from the theories of glycolysis to lactate metabolism, PPP and glucogenesis. It is intensively stressed that the high efficiency of glucose utilization is one of the most outstanding characteristics in TC cells. And targeting glucose metabolic process has got successive victories. The complicated lipid constituent forms the intricacy of metabolic changes in lipids. From lipid absorption to its utilization such as FAO and ketone bodies exploiting, there’s no doubt that TC cells have their own unique way of managing lipids. The amino acid and nucleotide metabolism is equally influential since the metabolic reprogramming should be regarded as a whole and each part is no way to neglect. And the TCA cycle as the center of multitudinous important metabolic pathways, has been studied with emphasis. Novel regulators, such as vitamins, hormones and ROS, have been demonstrated widely for their participation in all aspects of TC metabolism, and new regulators are under exploration. With the full view of TC metabolism, detecting and therapeutic strategies based on the reprogrammed metabolism in TC has developed quickly. ^18^F-FDG PET/CT, UHPLC-MS/MS and HPLC-TSQ-MS show high accuracy in several types of TC diagnosis. And chemotherapeutic drugs aiming at the altered metabolic process show great potential. Interestingly, some classical drugs used broadly in diseases of other systems, such as metformin and piperlongumine, may have new applications in TC treatment. Nanotherapeutics also have a promising future for the distinctive delivering and targeting character. From all above, research into the metabolic reprogramming of TC is quite worthy for its clinical value.

## Data Availability

No datasets were generated or analysed during the current study.

## Abbreviations

ATP: Adenosine 5’-triphosphate
TC: Thyroid cancer
PTC: Papillary thyroid cancer
FTC: Follicular thyroid cancer
MTC: Medullary thyroid cancer
ATC: Anaplastic thyroid cancer
PDTC: Poorly differentiated thyroid cancer
LA: Lactic acid
TCA cycle: Tricarboxylic acid cycle
OXPHOS: Oxidative phosphorylation
PPP: Pentose phosphate pathway
GLUT: Glucose transporter
SLC: Solute carrier family
ECM: Extracellular matrix
LncRNA: Long non-coding RNA
FTO: Fat-mass and obesity-associated protein
PTEN: Phosphatase and tensin homolog
SIRT: Sirtuin
Six1: Sineoculis homeobox homolog 1
E2: Estrogen
DNMT3B: DNA methyltransferases 3B
FAM111B: Family with sequence similarity 111 member B
PGC1α: Peroxisome proliferator-activated receptor γ coactivator 1α
AcCoA: Acetyl coenzyme A
HK2: Hexokinase 2
PFK1: Phosphofructokinase 1
PKM2: Pyruvate kinases type M2
ALKBH5: (AlkB homolog 5)
ANP32E: Acidic nuclear phosphoprotein 32 family member E
AMPK: AMP-activated protein kinase
miRNA: Micro RNA
PPAT: Phosphoribosyl pyrophosphate amidotransferase
LDH: Lactate dehydrogenase
MCT: Mono-carboxylate transporter
TME: Tumor microenvironment
G6P: Glucose 6-phosphate
R5P: Ribose 5-phosphate
GA3P: Glyceraldehyde 3-phosphate
F6P: Fructose 6-phosphate
G6PD: Glucose 6-phosphate dehydrogenase
BTG: Benign thyroid lesion in one lobe
ROS: Reactive oxygen species
6PGD: 6-Phosphogluconate dehydrogenase
LDL: Low-density lipoprotein
FA: Fatty acid
TG: Triglyceride
MUFA: Monounsaturated fatty acid
CPT: Carnitine palmitoyltransferase
CACT: Carnitine-acylcarnitine translocase
FAO: FA β-oxidation
AcAc: Acetoacetate
SREBP: Sterol regulator element binding protein
DTC: Differentiated thyroid cancer
FASN: Fatty acid synthase
ACC: Acetyl coenzyme A carboxylase
SCD: Stearoyl-CoA desaturase
PC: Phosphocholine
TSH: Thyrotropin
PLCD3: Phospholipase C Delta 3
BCAA: Branched-chain amino acid
*ABHD11-AS1*: ABHD11 antisense RNA 1
GLS1: Glutaminase 1
GLUD: Glutamate dehydrogenase
GCL: Glutamate-cysteine ligase
PHGDH: Phosphoglycerate dehydrogenase
SHMT: Serine hydromethyl transferase
PINK1: PTEN-induced kinase 1
MIEAP: Mitochondria-eating protein
ATG5: Autophagy-related gene 5
HIF-1: Hypoxia-inducible factor-1
YAP: Yes-associated protein
NF-κB: Nuclear factor-κB
IDH1: Isocitrate dehydrogenase 1
SDH: Succinate dehydrogenase complex
ERK: Extracellular signal-regulated kinase
STAT3: Signal transducer of activation 3
NDPK: Nucleoside diphosphate kinase
XCI: X-chromosome inactivation
XIST, 7-DHC: 7-Dehydrocholesterol
NIS: Sodium-iodine symporter
CRABP2: Cellular retinoic acid-binding protein 2
HMGB1: High-mobility group box 1
mtDNA: Mitochondrial DNA
mitoROS: Mitochondrial ROS
GPX4: Glutathione peroxidase 4
TH: Thyroid hormone
VEGF: Vascular endothelial growth factor
^18^F-FDG: ^18^F-fluorodeoxyglucose
PET: Position emission tomography
CT: Computed tomography
FDG-6-P: FDG-6-phosphatate
PSMA-11: Prostate-specific membrane antigen 11
^18^F-FGln: ^18^F-(2S,4R)4-fluoroglutamine
^18^F-TFB: ^18^F-tetrafluoroborate
^68^ Ga-DOTATATE: ^68^Ga-tetraazacyclododecane tetraacetic acid-DPhe1-Tyr3-octreotate
COF: Covalent organic framework
SPME: Solid-phase microextraction
UHPLC-MS/MS: Ultra-high-performance liquid chromatography-tandem mass spectrometry
DESI-MS: Desorption electrospray ionization mass spectrometry
HPLC-TSQ-MS: High-performance liquid chromatography-triple stage quadrupole-mass spectrometry
HT: Hashimoto's thyroiditis
TRIM26: Tripartite motif 26
DPP-IV: Dipeptidyl peptidase-IV
SGLT2: Sodium-glucose cotransporter 2
PDGFR: Platelet-derived growth factor receptor
AKR1C3: Aldo-keto reductase family 1 member C3
PGK1: Phosphoglycerate kinase 1
*PTCSC3*: Papillary thyroid cancer susceptibility candidate 3
JAZF1: Juxtaposed with another zinc finger gene 1
METTL16: Methyltransferase-like protein 16
STAG2: Stromal antigen 2
PIM1: Proviral integration site for Moloney murine leukemia virus 1
DON: 6-Diazo-5-oxo-l-norleucine
SHIN1: Serine hydroxymethyltransferase inhibitor 1
TRPV1: Transient receptor potential vanilloid 1
OCT4A: Octamer-binding transcription factor 4A
Cap-Au@MSN: Capsaicin loaded in Au@mesoporous silica nanoparticle
ACLY: ATP-citrate lyase
PDPR: Pyruvate dehydrogenase phosphatase regulatory
